# Can we rely on remote electrogram analysis to monitor long-term conduction system capture in left bundle branch area pacing?

**DOI:** 10.1093/europace/euag084

**Published:** 2026-05-05

**Authors:** Gianni Pastore, Lina Marcantoni, Matteo Spoladori, Francesco Deluca, Simone Valenza, Giorgio Porcelli, Graziano Boaretto, Carlotta Di Santo, Francesco Zanon, Sandra Marsiglia, Matteo Busacca, Franco Noventa

**Affiliations:** Department of Cardiology, Santa Maria Della Misericordia Hospital, Via Tre Martiri, Rovigo 140 45100, Italy; Department of Cardiology, Santa Maria Della Misericordia Hospital, Via Tre Martiri, Rovigo 140 45100, Italy; Department of Cardiology, Santa Maria Della Misericordia Hospital, Via Tre Martiri, Rovigo 140 45100, Italy; Department of Cardiology, Santa Maria Della Misericordia Hospital, Via Tre Martiri, Rovigo 140 45100, Italy; Department of Cardiology, Santa Maria Della Misericordia Hospital, Via Tre Martiri, Rovigo 140 45100, Italy; Department of Cardiology, Santa Maria Della Misericordia Hospital, Via Tre Martiri, Rovigo 140 45100, Italy; Department of Cardiology, Santa Maria Della Misericordia Hospital, Via Tre Martiri, Rovigo 140 45100, Italy; Department of Cardiology, Santa Maria Della Misericordia Hospital, Via Tre Martiri, Rovigo 140 45100, Italy; Department of Cardiology, Santa Maria Della Misericordia Hospital, Via Tre Martiri, Rovigo 140 45100, Italy; Department of Cardiology, Santa Maria Della Misericordia Hospital, Via Tre Martiri, Rovigo 140 45100, Italy; Department of Cardiology, Santa Maria Della Misericordia Hospital, Via Tre Martiri, Rovigo 140 45100, Italy; QUOVADIS no-profit Association, Padova, Italy

**Keywords:** Left bundle branch area pacing, Remote monitoring, Near-field electrogram, EGM, Conduction system pacing, Conduction system capture

## Background

Left bundle branch area pacing (LBBAP) is increasingly used to preserve physiological ventricular activation and prevent pacing-induced dyssynchrony.^1^ However, long-term maintenance of conduction system capture (CSC) is not always guaranteed. Lead micro- or macro-dislodgement or local tissue remodelling may result in a transition from conduction system to myocardial-only capture, with consequent widening of the paced QRS (pQRS).^2^ At present, CSC verification relies mainly on in-hospital 12-lead ECG assessment, which provides only intermittent evaluation and may fail to detect changes occurring between scheduled visits.^3,4^ Remote monitoring (RM) systems routinely transmit intracardiac electrograms (EGMs), allowing continuous observation of pacing-related electrical activity under real-world conditions,^5^ thereby providing the opportunity to detect subtle changes in pacing morphology. The purpose of this preliminary data analysis was to evaluate whether changes in NF-EGM morphology and duration, transmitted through RM, can closely mirror pQRS modifications, potentially reflecting CSC status in LBBAP patients.

## Methods

**Figure 1:**
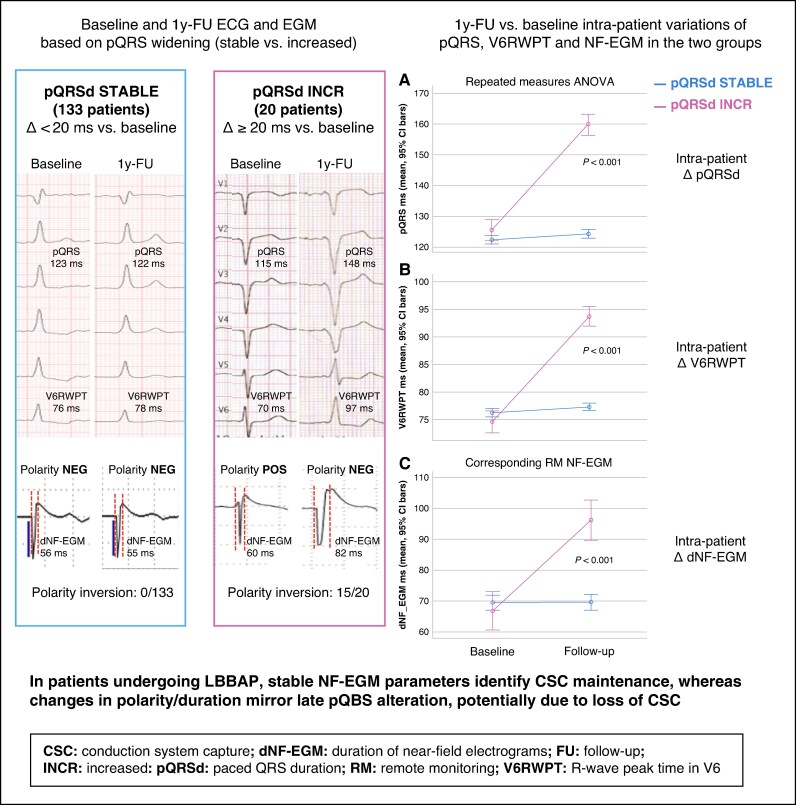
(Left) Baseline and 1y-FU ECG and EGM based on pQRS widening (stable vs. increased); (Right) 1y-FU vs. baseline intra-patient variations of pQRS, V6RWPT and NF-EGM in the two groups.

We retrospectively analysed 153 patients who underwent LBBAP with confirmed CSC at implantation and immediate RM activation. Patients were selected from a larger LBBAP population implanted at Santa Maria della Misericordia Hospital, Rovigo, Italy, based on the availability of matched RM transmission with 12-lead ECGs performed at implantation, 1-year follow-up (1y-FU) visits, or urgent visits. All implantations were performed according to the European Heart Rhythm Association Consensus criteria for conduction system pacing.^6,7^ Both lumenless leads (LLL) and stylet-driven leads (SDL) were utilized based on operator preference. Twelve-lead ECGs were analysed at baseline, immediately post-implant, and at 1y-FU. Paced QRS duration (pQRSd) and stimulus-to–R-wave peak time in lead V6 (V6RWPT) were measured. Based on pQRSd variations at 1y-FU, patients were classified into two groups: PQRS-STABLE (ΔpQRSd <20 ms) and pQRS-INCR (ΔpQRSd ≥20 ms). NF-EGMs were derived from the bipolar tip-to-ring configuration of the LBBAP lead and analysed using standardized gain settings. NF-EGMs were classified based on rapid deflection duration (dNF-EGM: interval from onset to transition towards slower activation, marked by the appearance or delay of notching), and the polarity inversion of the initial rapid deflection relative to the isoelectric line.^8,9^ Illustrative cases of ECG and EGM at baseline and 1y-FU are reported in *Figure 1*. Clinical, procedural, and device-related variables were collected. Group comparisons and multivariable logistic regression were performed to identify factors associated with pQRS widening.

## Results

At 1y-FU, 133 patients (87%) remained pQRS-STABLE, while 20 patients (13%) met criteria for pQRS-INCR. Compared with the pQRS-STABLE group, pQRS-INCR patients exhibited significantly longer pQRS (159.8 ± 9.7 ms vs. 124.3 ± 7.5 ms; *P* < 0.001) and V6RWPT (93.7 ± 4.7 ms vs. 77.3 ± 3.9 ms; *P* < 0.001). In parallel, a significant prolongation of the dNF-EGM in the pQRS-INCR group from 66.8 ± 11.7 ms to 96.2 ± 19.1 ms (*P* < 0.001), accompanied by polarity inversion in 75% of cases (15/20) was observed. Neither a significant change in dNF-EGM duration (+20 ms) nor polarity inversion was observed in pQRS-STABLE patients. The sensitivity and specificity for predicting pQRS-INCR using dNF-EGM change or polarity inversion were 0.75 and 0.964, respectively. One-year FU vs. baseline intra-patient variation of pQRS, V6RWPT, and dNF-EGM in both groups are shown in *Figure 1*. Only 2 (10%) patients with pQRS widening showed radiological evidence of macro-dislodgement (from the initial deep intra-septal site towards a right ventricular endocardial position). Therefore, in the remaining 18 patients, the likely cause was lead micro-dislodgment, suggesting that subtle mechanical or tissue-related factors may underlie most cases of CSC instability. LBBAP was performed using LLL in 86 patients (57%) and SDL in 66 (43%). Use of stylet-driven leads emerged as an independent predictor of pQRS widening (OR 3.0, 95% CI 1.1–8.7, *P* = 0.038).

## Discussion

This preliminary data analysis demonstrates a consistent association between surface ECG markers of CSC loss and specific NF-EGM waveform changes detected through routine RM systems. Importantly, these findings were obtained using real-world device diagnostics rather than dedicated evoked-response sensing, supporting their pragmatic clinical relevance. NF-EGM modifications should not be interpreted as direct recordings of conduction system potentials. Rather, they likely reflect an integrated manifestation of altered local activation dynamics that occur when ventricular activation shifts from rapid conduction system propagation to slower myocardial conduction. In this context, prolongation and morphological modification of the NF-EGM rapid deflection appear to mirror the loss of physiological activation observed on surface ECG. Previous studies have demonstrated the diagnostic value of EGM morphology assessment during in-hospital testing for conduction system pacing.^7,8^ Our findings extend these observations to long-term remote monitoring, suggesting that routinely transmitted EGMs may provide clinically meaningful information on CSC stability without requiring additional hardware or patient interaction. This pilot study should be considered hypothesis-generating. We neither propose diagnostic cut-offs nor claim that EGM changes anticipate ECG findings. Rather, we suggest feasibility and signal consistency, providing a foundation for future prospective studies and the development of automated EGM pattern-recognition tools, in a setting of data integration and interoperability extended to remote monitoring.^10^

## Limitation section

This was a retrospective, hypothesis-generating analysis restricted to patients with temporally matched RM transmissions and 12-lead ECGs, which may introduce selection bias and limits generalizability. NF-EGM assessment was limited to transmissions with visually discernible signals, and pacing artefact/amplifier saturation may have affected signal interpretability. Measurements of dNF-EGM duration and polarity were performed retrospectively without formal blinding or inter-observer variability assessment. In addition, systematic testing across pacing outputs or unipolar configurations was not performed; therefore, bipolar NF-EGM changes should be interpreted as indirect correlates of altered ventricular activation rather than direct markers of left bundle capture.

## Conclusions

In patients undergoing LBBAP, stable NF-EGM patterns indicate maintained physiological pacing. Changes in NF-EGM morphology and duration detected through RM can serve as markers of CSC instability. Remote NF-EGM analysis may support future strategies for monitoring conduction system pacing.

F.Z. received speaker’s fees from Abbott Medical, Biotronik, Boston Scientific and Medtronic. F.N. received fees for statistical and methodological consulting from Abbott Medical. The other authors declared no conflicts of interest.

## Data Availability

Data available on request due to privacy/ethical restrictions, Basic, Share upon Request. The data that support the findings of this study are available on request from the corresponding author, [GP]. The data are not publicly available due to [restrictions: their containing information that could compromise the privacy of research participants].
